# Influence of Biomedical Factors on the Five Viscera Score (FVS) on Middle-Aged and Elderly Individuals: Application of Structural Equation Modeling

**DOI:** 10.1155/2015/687015

**Published:** 2015-10-01

**Authors:** Taro Tomura, Kouichi Yoshimasu, Shunji Sakaguchi, Kanami Tsuno, Shigeki Takemura, Nobuyuki Miyai, Kazuhisa Miyashita

**Affiliations:** ^1^Acupuncture-Moxibustion and Sports Trainer Science, Kansai University of Health Sciences, 2-11-1 Wakaba, Kumatori, Sennan, Osaka 590-0482, Japan; ^2^Department of Hygiene, School of Medicine, Wakayama Medical University, 811-1 Kimiidera, Wakayama 641-8509, Japan; ^3^School of Health and Nursing Science, Wakayama Medical University, 580 Mikazura, Wakayama 641-0011, Japan

## Abstract

The five viscera score (FVS) is a diagnostic scale for traditional Chinese medicine (TCM). The purposes of current study are to elucidate the characteristics of FVS obtained from middle-aged to elderly individuals and to investigate the validity of FVS using biological medical data of middle-aged and elderly individuals. Structural equation modeling (SEM) was used to conduct assessments between FVS and medical data. Eighty men and 99 women participated in this study, whose mean ages (SD) were 58 ± 7 years in both genders showing no significant difference. FVS of women was significantly higher than that of men in the spleen of the 50s (*P* = 0.019) and liver of the 60s age group (*P* = 0.030). By SEM, the following biomedical factors were found to influence viscera: gender, diastolic blood pressure, and HDL-C for the liver; GLU, GOT, and *γ*-GTP for the spleen; age, BMI, and HCRP for the lungs; and HbA1c and creatinine clearance for the kidneys. These results provide objective evidence that FVS can be used for TCM diagnosis in middle-aged and elderly individuals.

## 1. Introduction

Acupuncture therapy is currently recognized as a complementary and alternative medicine (CAM) that is performed around the world, especially in East Asia [1]. In order to investigate its efficacy, there have been several intervention studies involving specific diseases [2–4]. However, because the classification of symptoms in traditional Chinese medicine (TCM) depends on the clinical experience of the TCM practitioner, the diagnosis and acupuncture points often differ even for the same patient or for patients with the same disease [1, 5, 6]. Therefore, the treatment effect is the primary focus of these investigations and treatment strategies are rarely investigated [2–4]. Furthermore, these treatment effects may be affected by inconsistent treatment strategies. One of the reasons for this is that there are very few diagnostic methods that are based on evidence in TCM [7]. Establishing an effective diagnostic method is essential in order to resolve this issue. Although the benefits of TCM may be eliminated through the creation of a standardized diagnostic method that elucidates the effects of acupuncture therapy as effective acupuncture points vary according to each individual's physical constitution, the development of acupuncture therapy research and its application can be anticipated by developing a common diagnostic scale.

That is why we developed a “five viscera score (FVS)” as a diagnostic scale for TCM. Items for this scale were statistically selected from the symptoms of the five viscera (liver, heart, spleen, lungs, and kidneys) that were extracted from key TCM literature from the past 2000 years [8–10]. These “five viscera” refer to the viscera in oriental medicine and are known to be the center of vital activities, possessing both morphological and functional concepts [11]. In general, using both classical test theory (CTT), such as Cronbach's alpha, and item response theory (IRT), which can remove the effects from the original population that developed the scale, increases the validity of the developed scale [12, 13]. We therefore tested FVS with both CTT and IRT and have demonstrated its effectiveness [9, 10, 14]. However, common limitations among these studies were that they included very few women in the study population and did not assess middle-aged and elderly individuals. The purpose of this study is twofold: first to elucidate the characteristics of FVS obtained from middle-aged to elderly individuals and second to investigate the validity of FVS using biological medical data such as blood samples from middle-aged and elderly individuals as external criteria.

Structural equation modeling (SEM) was used to conduct assessments between FVS and medical data. SEM is a method that uses a path diagram to analyze the relationship between various factors that exist behind the observed data [15]. As mentioned previously, treatment strategies have not been sufficiently investigated in the previous intervention studies of acupuncture since there were no appropriate approaches that could evaluate concepts of TCM, such as the five viscera (termed “latent variable” in SEM). SEM, which can simultaneously evaluate relationships between latent variables (five viscera) and observed variables (FVS items and medical data), is one optimal technique for investigating diagnostic methods in TCM. However, there are very few studies that have investigated diagnostic scales of TCM using SEM [16, 17], and there are no studies for the five viscera except ours [10].

## 2. Materials and Methods

### 2.1. Study Subjects and Implementation Method

Subjects included 212 individuals aged 40–65, who were residents of Wakayama Prefecture in Japan, underwent specific health checkups for metabolic syndrome, wished to participate in this study, and were able to attend the study site without assistance. Specific health checkups refer to a medical examination of individuals aged 40–74 who have public health insurance coverage. This study was conducted in August 2012 for 6 days. Consent was ultimately obtained from 189 individuals (89.2%), and the investigation was conducted in 179 individuals (84.4%). Ten individuals were excluded due to incomplete test values.

### 2.2. Ethical Considerations

This study was approved by the Genetic Analysis Research Ethics Committee at Wakayama Medical University (number 92) and by the Ethics Committee of Kansai Vocational College of Medicine (number H25- 01). The purpose of this study was explained to the subjects and only those who signed the consent form were allowed to participate. All individuals were assigned an ID and made anonymous. Test results were consolidated by a third party.

### 2.3. The Five Viscera Score

FVS is composed of viscera symptoms as defined in TCM [8] and uses a self-report questionnaire format that asks questions concerning physical condition from the previous month [18]. These symptoms were selected through an explanatory factor analysis and considered to be affected by the viscera as latent factors. Naming of each factor was determined based on many TCM practitioners' opinion [10, 14, 18]. In a previous study, we utilized FVS that contained items that were difficult to answer (high item difficulty) [14]. We modified this to be more suitable for clinical studies and the general public to reduce the burden on the respondent. The validity of this FVS has been confirmed by reducing the number of items [18]. The following items from the previous version were removed: “I have migraine headaches (headaches)” in the liver category, “I sigh often” in the heart, “I do not have much energy in the morning” in the spleen, “I get the hiccups” in the lungs, and “I feel lethargic to the point where I have to lie down” in the kidneys. A total of 15 items composed the new FVS version, which was used in the present study (Table 1). Cronbach's alpha coefficient showed almost the same value compared to the previous version of FVS (liver (*r* = 0.77), heart (*r* = 0.82), spleen (*r* = 0.82), lungs (*r* = 0.64), kidneys (*r* = 0.79), and overall (*r* = 0.91)).

The items that were ultimately removed were selected by referring to previous studies that used both CTT and IRT, as described below. Items with the lowest factor loading within each subscale according to factor analysis [9] and items that have low power to discriminate the presence or absence of symptoms and have exceedingly high difficulty within each subscale according to IRT [14] were deleted when creating the FVS. The new FVS also consisted of a 5-point item Likert scale for choices: “none of the time (0 points),” “a little of the time (1 point),” “some of the time (2 points),” “most of the time (3 points),” and “all of the time (4 points).” The subscale score was a total of the item scores (0 to 12 points), and the viscera with the highest score balance were determined as the TCM diagnosis “Zheng,” which refers to the TCM syndrome. By removing items that contribute very little to the subscale, the characteristics of each viscus became clearer in the modified FVS, and its validity for health evaluation also increased [18].

### 2.4. Medical Data

To investigate the relationship between health and FVS, which is a diagnostic scale of TCM, we used medical data including body mass index (BMI), blood pressure, and blood and urine test values. Subjects provided fasting venous blood and urine samples in the morning on the health checkup day. Test variables in the subjects' blood and urine include triglyceride (TG) (mg/dL), low-density lipoprotein cholesterol (LDL-C) (mg/dL), high-density lipoprotein cholesterol (HDL-C) (mg/dL), glucose (GLU) (mg/dL), hemoglobin A1c (HbA1c) (%), uric acid (UA) (mg/dL), glutamic oxaloacetic transaminase (GOT, AST) (IU/L), glutamic pyruvic transaminase (GPT, ALT) (IU/L), *γ*-glutamyl transpeptidase (*γ*-GTP) (IU/L), red blood cell (RBC) (×10^4^/uL), hematocrit (HCT) (%), systolic blood pressure (mmHg), diastolic blood pressure (mmHg), high-sensitivity C-reactive protein (HCRP) (mg/dL), urinary protein (mg/dL), albumin/creatinine ratio (ACR) (mg/gCr), and creatinine clearance (Ccr) (mL/min). The analyses were conducted at Osaka Kessei Research Laboratories, Inc.

### 2.5. Statistical Analysis

#### 2.5.1. Structural Equation Modeling (SEM)

**Figure 1:**
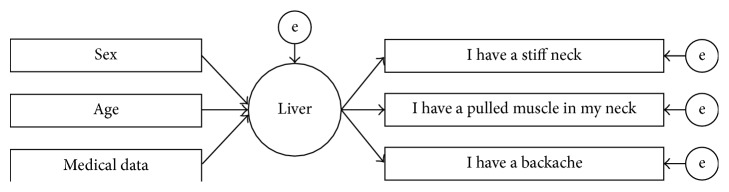
MIMIC model of the liver by the structure equation modeling. The square of the figure is an observation variable, and the central circle is a latent variable. “e” means an error. For an example, we cast sex (male 0, female 1) and age as well as medical data into the left side adopted by exploratory model specialization across the liver which is a latent capacity variable. Subscale items of the FVS are set in the right side.

Amos 19, a program for SEM by IBM, was used. All linkage strengths estimated in the path diagram (path coefficient) were approximation of the standardised partial regression coefficient with a mean of 0 and variance of 1; thus the results were not affected by units. The path diagram was constructed using the multiple indicator multiple cause model (MIMIC model) with the latent variable of five viscera positioned between observed medical data and FVS items (Figure 1). This MIMIC model is commonly used in SEM, and we used this model where the latent variable “viscera” are defined by multiple medical data, and these viscera influence a number of other observed “FVS items.” Furthermore, in order to determine data that significantly affect viscera from the numerous pieces of medical data, we conducted a “model specification search” that determines the most suitable combination by repeating exploratory confirmative factor analysis [15]. As an example (see Figure 1), the latent variable liver is interposed between medical data, gender for adjustment (men 0, women 1), and age that are input from the left side and FVS subscale items on the right side.

In addition, coefficient of determination (*R* ^2^), goodness of fit index (GFI), adjusted GFI (AGFI), comparative fit index (CFI), and root mean square error of approximation (RMSEA) were used as indices to determine the suitability of the overall model. GFI, AGFI, and CFI that are ≥0.90 and RMSEA that is ≤0.10 were deemed satisfactory.

#### 2.5.2. Other Statistical Analyses

Since there is a gender difference in FVS [14], the Mann-Whitney *U* test was used to compare between men and women, and Spearman's rank correlation coefficient was used (SPSS 21; IBM). Differences of age distributions between the genders were evaluated by chi-square test. Although these statistical tests are nonparametric, mean values as well as their standard deviations (SD) were presented in tables to examine the floor and ceiling effects. For all statistical calculations, significant level was set at <0.05. However, *P* values less than 0.10 were regarded as statistically marginally or slightly significant since current purpose is to detect a broad range of biomedical factors (including even nonsignificant factors) that make any influence on the viscera.

## 3. Results

### 3.1. Characteristic of Subjects

There were 80 men and 99 women participating in this study. Subject characteristics as well as medical data input into SEM are shown in Table 2. Mean ages (± SD) of men and women were both 58 ± 7 years, showing no significant difference. Age distributions between the genders were not also significantly different (*P* = 0.83).

### 3.2. FVS according to the Generation

Table 3 shows the FVS (overall and by age group) that was compared between men and women. Symptoms of the heart and spleen of men in their 40s and those of liver of men in their 60s had a large variation, showing that their standard deviations were larger than the mean values (floor effect). SD of kidney in 40s males was also wide though not reaching floor effect. In addition, FVS was greater in women compared to men in the 50s and 60s age groups (excluding 40s). Statistical significance was observed in the spleen of the 50s (*P* = 0.019) and liver of the 60s age group (*P* = 0.030), and similar tendency was also observed in the kidneys of 60s age group (*P* = 0.075) though not reaching statistically significant level. In the entire sample, women also showed greater FVS excluding the lungs, and significant differences were observed in the liver (*P* = 0.019) and spleen (*P* = 0.032).

### 3.3. Exploratory Model Specification by the Structure Equation Modeling (SEM)

Using SEM, a model specification search was carried out for each viscus in order to identify observed variables (medical data) on the left side that significantly influence the latent variable (viscera). Medical data that were input for each subscale of FVS created 262,144 combinations for analysis, and the most suitable model was used.

#### 3.3.1. Effects on Viscera from Biomedical Factors

Table 4 is a summary of the path coefficients as well as suitability indices of each model, showing path coefficients from specified medical data to the viscera with gender and age controlled.

The coefficients of determination of the model were 0.182 for the liver, 0.026 for the heart, 0.348 for the spleen, 0.155 for the lungs, and 0.092 for the kidneys, indicating that the spleen had the highest value. Medical data that had significant (or slightly significant) influence on viscera were gender, HbA1c, diastolic blood pressure, HCRP, and HDL-C in the liver; age in the heart; gender, age, GLU, GOT, *γ*-GTP, BMI, diastolic blood pressure, and HCRP in the spleen; age, BMI, and HCRP in the lungs; and HbA1c, creatinine clearance, and HCRP in the kidneys.

Furthermore, in order to elucidate items that especially affect the viscera, we selected items from the above list with high path coefficients that did not overlap other viscera (if there was an overlap, the item with a greater path coefficient was selected) and determined them to be distinctive items. This procedure was conducted to extract factors that make influences on the viscera most strongly. In other words, such items are considered to be well representative of the viscera's characteristics. Since the path coefficients were standardized as mentioned above, they were comparable to each other. Distinctive items that influenced the viscera as well as their path coefficients were gender (0.273), diastolic blood pressure (0.178), and HDL-C (−0.131) for the liver; GLU (−0.223), GOT (0.312), and *γ*-GTP (−0.290) for the spleen; age (−0.252), BMI (0.188), and HCRP (0.181) for the lungs; and HbA1c (−0.193) and creatinine clearance (0.153) for the kidneys. No items were selected for heart.

#### 3.3.2. Effects on FVS from Viscera

The path coefficients from the latent variables (viscera) to the FVS items on the right side were as follows: for the liver, “I have a stiff neck” (0.857), “I have a pulled muscle in my neck” (0.892), and “I have a backache” (0.612); for the heart, “I worry about many things” (0.883), “I worry frequently” (0.942), and “I have a lot on my mind and am not able to enjoy anything” (0.716); for the spleen, “I am fatigued and this is not alleviated by anything” (0.802), “I have to lie down due to fatigue” (0.719), and “My body feels heavy” (0.870); for the lungs, “My stomach rumbles” (0.690), “I feel hungry constantly” (0.640), and “I have a runny nose” (0.271); and for the kidneys, “I am absent minded” (0.858), “I am not energetic” (0.752), and “My memory has deteriorated” (0.513). With the exception of “I have a runny nose” in the lungs, the path coefficients were greater than 0.5, indicating that FVS items are strongly influenced by latent viscera.

## 4. Discussion

To test the validity of FVS in middle-aged and elderly individuals, we investigated the characteristics of FVS as well as the relationship between medical data (external criteria) and FVS using SEM with the MIMIC model. The results indicated that the characteristics showed gender differences, consistent with previous findings, and SEM showed a lot of the medical data influencing the viscera.

FVS by age group showed that women in their 50s and 60s had higher scores, and this was consistent with a previous study that reported the relationship between different genders in 594 young to middle-aged individuals [14]. For FVS in the 40s age group, men had a greater score. However, the gender difference was not significant, as the sample size was small and there was large variation.

Concerning subjective health status, the Medical Outcome Study Short-Form 36-Item Health Survey version 2 (SF-36), an evaluation scale that is used globally, and Japanese national standard values of this SF-36 have been reported [19]. Here, all subscales of health status in women show lower value than the national standards and are also lower compared to men. Moreover, comparisons by age group in individuals aged 40–69 also demonstrated that women have lower scores in many of the subscales related to health compared to men. Based on the fact that subjective health status in women was poorer than men in this study and through our previous report involving young to middle-aged individuals in which the positive association between FVS and SF-36 was observed [14], it was considered that FVS is an appropriate and valid scale to demonstrate subjective health status.

SEM showed that many of the biomedical factors influenced viscera with the most distinctive items being gender, diastolic blood pressure, and HDL-C for the liver; GLU, GOT, and *γ*-GTP for the spleen; age, BMI, and HCRP for the lungs; and HbA1c and creatinine clearance for the kidneys. Gender and diastolic blood pressure positively influenced the liver; however, perhaps due to the narrow age range (middle-aged to elderly) of the subjects, diastolic blood pressure and age were not correlated with each other (men: *r* = −0.153, women: *r* = −0.002; data not shown). In addition, HDL-C, which is related to atherosclerosis, had a negative influence on the liver. Since the heart represents mental activity in TCM [11], lack of the direct influence of biomedical factors examined in the routine health checkup on mental status was quite natural. Fasting blood glucose levels (GLU) had a negative influence on the spleen, indicating that blood sugar may be related to the spleen. Moreover, GOT and *γ*-GTP, which are both related to the liver (digestive organ), had positive and negative influence, respectively, on the spleen symptoms. Age had a negative influence on the lungs, indicating stronger symptoms in the younger age group, and HCRP has a positive influence. These facts suggest that this was an inflammatory response to conditions such as infections. In addition, the elevation in BMI indicated obesity, which is a risk factor of many kinds of lifestyle-related diseases, and this was also thought to promote inflammation. HbA1c had a negative influence on the kidneys, suggesting that blood sugar and red blood cells were involved in the mechanism. In addition, creatinine clearance had a positive influence. These items had path coefficients that were not very high, while they included marginally significant influences.

Because the viscera in TCM are comprehensive concepts that not only possess morphological aspects, but also entail functional aspects, the influence from medical data to the viscera is restrictive. Furthermore, gender and age were both input compulsorily, thereby lowering the coefficient of determination and the goodness of fit of the model.

Most of the items in FVS are unidentified complaints. In both Western medicine and TCM, unidentified complaints are crucial to disease prevention; thus it was clinically important that there was an association between the medical data based on Western medicine with TCM. Moreover, it is noteworthy that the specific related medical data fit the descriptions of viscera characteristics in TCM textbooks as shown below [11]. The liver controls mental activity and stores blood. Abnormalities in the liver lead to the reversal of blood and impairment of distribution. Therefore, the liver is considered to be associated with the factors related to pliability of blood vessels, such as diastolic blood pressure and HDL-C. The spleen controls digestion and absorption of foods and drinks. Therefore, the spleen is considered to be associated with digestive functions in liver or pancreas such as GLU, GOT, and *γ*-GTP.

The lung systemically circulates Qi and bodily fluids (such as saliva and tears). Stagnation leads to the development of edema. In addition, invasions by the common cold (i.e., external pathogens (wind)) are likely to occur. Hence, the lung is considered to be associated with factors related to obesity and inflammation, such as BMI or HCRP, which might reflect the spleen disorder rather than lung disorder by the TCM criteria. However, since FVS is a newly created scale as mentioned earlier, some of which might be inconsistent with the TCM literature. The kidney is supplemented by the energy created from foods and drinks. In addition, it eliminates liquids that are no longer necessary as urine. Therefore, the kidney is considered to be associated with factors related to blood sugar or renal function such as HbA1c or creatinine clearance. Because kidney and spleen dysfunction is attributed to diabetes, it is unclear why GLU and HbA1c were negatively associated with spleen and kidney as well as the discrepancy between GOT and *γ*-GTP observed in the spleen. These may be partially due to the cross-sectional characteristics of the current study. Prospective clinical cohort study will be necessary to resolve this inconsistency. However, as mentioned earlier, it should be noted that the FVS is created based on the TCM theory and does not reflect the clinical characteristics of corresponding anatomical internal organs.

In a TCM study of hypertension, Wu et al. discovered two biomarkers from diagnostic standards of multiple TCM syndromes and reported that these are useful for classifying and discriminating TCM syndrome [20]. Here, we did not restrict the study to a specific disease but investigated multiple biomarkers related to one TCM diagnostic scale from various angles. Therefore, this study is significant in that we have discovered distinctive relationships. This study also revealed that all FVS items on the right side of viscera as latent variable, except for “I have a runny nose” in the lungs, were strongly influenced by viscera. These results signify that FVS can be used for TCM diagnosis in middle-aged and elderly individuals.

In a study of TCM diagnosis presented in 2012, Wang et al. reported that SEM is an effective method [16]. SEM is considered to be useful for TCM study since it allows concepts of TCM such as viscera to be evaluated as latent variables. In this study, we showed that specific biomedical factors significantly influenced each of the viscera in TCM, which was also consistent with former TCM interpretations. Therefore, current findings added scientific significance to the diagnosis of TCM, which had been regarded as diagnostic measure through clinical experiences. SEM should be further utilized in studies of diagnostic scales that determine treatment strategies for TCM.

In TCM, patients are typically diagnosed in a comprehensive manner using four methods: “observation,” “auscultation and smell,” “inquiry,” and “palpation” [21]. Therefore, it is not definitive whether or not diagnosis can be made with FVS inquiries alone. Nonetheless, if the reproducibility of the study is increased through additional studies using FVS, then FVS may become the standard for inquiry. FVS is a scale that adds objectivity to the diagnoses that have previously been affected by the TCM provider's subjective evaluation. FVS can be used in all TCM and CAM practices that perform diagnoses based on the state of the viscera in TCM. Furthermore, FVS may profit from combining its usage with previously investigated methods for TCM diagnosis [16, 22, 23]. One of the limitations of this study is that because the study was a cross-sectional design, we were not able to investigate over time whether those who had biomedical factors related to FVS would develop an illness linked with the FVS. Currently, we are continuing the study in residents in the same area. Findings from this cohort study will be reported in the future.

## 5. Conclusion

We found gender differences of FVS and identified several biomedical factors that significantly influenced viscera by application of structural equation modeling. The current findings suggest that FVS can be useful for TCM diagnosis incorporated with Western medicine in middle-aged and elderly individuals.

## Figures and Tables

**Table 1:** Validity of the revised version of FVS (*n* = 594).

Subscale	Item number	Item	Factor loading	*a* (SE)	Mean *b*	Cronbach's *α*
Liver						0.79
	Q1	I have a stiff neck	0.82	2.11 (0.11)	0.11	
	Q2	I have a pulled muscle in my neck	0.81	2.57 (0.14)	0.55	
	Q3	I have a backache	0.49	0.77 (0.06)	1.40	

Heart						0.83
	Q4	I worry about many things	0.83	2.40 (0.12)	0.40	
	Q5	I worry frequently	0.76	1.84 (0.10)	0.50	
	Q6	I have a lot on my mind and am not able to enjoy anything	0.57	1.09 (0.07)	1.19	

Spleen						0.79
	Q7	I am fatigued and this is not alleviated by anything	0.69	1.76 (0.09)	0.25	
	Q8	I have to lie down due to fatigue	0.60	0.93 (0.06)	0.61	
	Q9	My body feels heavy	0.55	1.62 (0.08)	0.33	

Lung						0.63
	Q10	My stomach rumbles	0.74	1.45 (0.08)	0.70	
	Q11	I feel hungry constantly	0.58	1.01 (0.07)	1.29	
	Q12	I have a runny nose	0.41	0.61 (0.05)	1.30	

Kidney						0.78
	Q13	I am absent minded	0.58	1.70 (0.09)	0.80	
	Q14	I am not energetic	0.53	1.45 (0.08)	0.98	
	Q15	My memory has deteriorated	0.49	1.01 (0.07)	1.03	

FVS: five viscera score, “*a*”: item discrimination, and SE: standard error. Mean “*b*” is the average of item difficulty values.

Item discrimination represents the ability of an item to differentiate the subjects; Overall Cronbach's *α* = 0.89.

Values of factor loading, item discriminability, and the mean item difficulty were used for developing original FVS.

Cronbach's alpha coefficient was recalculated for revised version.

**Table 2:** Biomedical characteristics of respondents.

Item	Male (*N* = 80)	Female (*N* = 99)	*P* value
	*n* (%)	*n* (%)	
Age (y)			
40–49	12 (15.0)	12 (12.1)	
50–59	20 (25.0)	27 (27.3)	
60–65	48 (60.0)	60 (60.6)	
Whole subjects mean (SD)	58.1 (7.2)	58.1 (6.5)	

	Male (*N* = 80)	Female (*N* = 99)	*P* value
	Mean (SD)	Mean (SD)	

Medical data			
BMI (kg/m^2^)	23.7 (2.8)	22.0 (4.2)	<0.001
TG (mg/dL)	150.8 (105.4)	110.0 (56.3)	0.003
HDL-C (mg/dL)	59.1 (14.5)	68.7 (12.6)	<0.001
LDL-C (mg/dL)	119.5 (31.8)	126.6 (30.2)	0.087
GLU (mg/dL)	102.1 (18.5)	93.1 (14.7)	<0.001
HbA1c (%)	5.2 (0.6)	5.1 (0.5)	0.545
UA (mg/dL)	6.1 (1.5)	4.3 (1.0)	<0.001
GOT (IU/L)	25.7 (13.4)	22.3 (5.7)	0.014
GPT (IU/L)	27.1 (13.6)	19.2 (6.5)	<0.001
*γ*-GTP (IU/L)	60.5 (130.0)	29.5 (81.0)	<0.001
Urinary protein (mg/dL)	12.3 (11.4)	10.4 (1.8)	0.774
RBC (×10^4^/uL)	487.3 (40.1)	440.7 (28.4)	<0.001
HCT (%)	46.2 (3.4)	41.2 (3.0)	<0.001
Systolic blood pressure (mmHg)	129.7 (17.1)	119.1 (15.7)	<0.001
Diastolic blood pressure (mmHg)	76.3 (11.3)	69.2 (9.3)	<0.001
Creatinine clearance (Ccr) (mL/min)	83.0 (16.7)	85.8 (15.4)	0.325
Albumin/creatinine ratio (mg/gCr)	19.3 (56.2)	13.7 (15.5)	0.002
HCRP (mg/dL)	0.042 (0.057)	0.042 (0.094)	0.230

Body mass index (BMI), triglyceride (TG) (mg/dL), low-density lipoprotein cholesterol (LDL-C) (mg/dL), high-density lipoprotein cholesterol (HDL-C) (mg/dL), glucose (GLU) (mg/dL), hemoglobin A1c (HbA1c) (%), uric acid (UA) (mg/dL), glutamic oxaloacetic transaminase (GOT, AST) (IU/L), glutamic pyruvic transaminase (GPT, ALT) (IU/L), *γ*-glutamyl transpeptidase (*γ*-GTP) (IU/L), red blood cell (RBC) (×10^4^/uL), hematocrit (HCT) (%), systolic blood pressure (mmHg), diastolic blood pressure (mmHg), high-sensitivity C-reactive protein (HCRP) (mg/dL), urinary protein (mg/dL), albumin/creatinine ratio (ACR) (mg/gCr), and creatinine clearance (Ccr) (mL/min). As for medical data, significant differences were observed for all except LDL-C, HbA1c, urinary protein, creatinine clearance, and HCRP by Mann-Whitney *U* test.

**Table 3:** FVS according to sex and age class.

Generation	Subscale	*n*	Male	*n*	Female	*P* value
			Mean (SD)		Mean (SD)	
Whole subjects	Liver	80	3.2 (2.8)	99	4.5 (3.4)	0.019
	Heart	80	2.9 (2.8)	99	3.2 (2.3)	0.125
	Spleen	80	3.3 (2.7)	99	4.0 (2.2)	0.032
	Lung	80	2.3 (1.8)	99	2.1 (1.5)	0.466
	Kidney	80	2.7 (2.4)	99	2.8 (1.8)	0.406

40–49	Liver	12	3.4 (2.2)	12	3.3 (2.5)	0.887
	Heart	12	4.2 (4.4)	12	3.1 (2.2)	0.932
	Spleen	12	4.2 (4.4)	12	3.7 (2.1)	0.713
	Lung	12	3.2 (1.9)	12	2.3 (1.5)	0.291
	Kidney	12	3.5 (3.3)	12	1.8 (1.3)	0.266

50–59	Liver	20	4.3 (2.7)	27	5.9 (3.5)	0.186
	Heart	20	3.2 (2.1)	27	3.5 (1.9)	0.499
	Spleen	20	3.7 (1.9)	27	5.1 (1.8)	0.019
	Lung	20	2.6 (1.5)	27	2.4 (1.6)	0.584
	Kidney	20	3.4 (2.1)	27	3.1 (1.2)	0.583

60–65	Liver	48	2.7 (2.9)	60	4.1 (3.4)	0.030
	Heart	48	2.5 (2.5)	60	3.2 (2.5)	0.129
	Spleen	48	3.0 (2.5)	60	3.6 (2.3)	0.174
	Lung	48	2.0 (1.9)	60	1.9 (1.5)	0.940
	Kidney	48	2.3 (2.1)	60	2.9 (2.0)	0.075

Mann-Whitney *U* test.

**Table 4:** The effects on viscera from biomedical factors.

Subscale	Item	Path coefficient	*P* value	*R* ^2^	GFI	AGFI	RMSEA
Liver	Sex^*^	0.273	<0.001	0.182	0.898	0.830	0.117
	Age	−0.041	0.577				
	HbA1c	−0.179	0.015				
	Diastolic blood pressure^*^	0.178	0.016				
	HCRP	0.157	0.033				
	HDL-C^*^	−0.131	0.075				

Heart	Sex	0.085	0.269	0.026	0.975	0.924	0.086
	Age	−0.136	0.079				

Spleen	Sex	0.172	0.012	0.348	0.811	0.716	0.174
	Age	−0.129	0.059				
	GLU^*^	−0.223	0.001				
	GOT^*^	0.312	<0.001				
	*γ*-GTP^*^	−0.290	<0.001				
	BMI	0.172	0.012				
	Diastolic blood pressure	0.156	0.023				
	HCRP	0.131	0.054				

Lung	Sex	−0.024	0.789	0.155	0.967	0.940	0.042
	Age^*^	−0.252	0.008				
	LDL-C	0.150	0.101				
	BMI^*^	0.188	0.042				
	HCRP^*^	0.181	0.050				

Kidney	Sex	−0.004	0.955	0.092	0.900	0.821	0.142
	Age	0.016	0.841				
	HbA1c^*^	−0.193	0.016				
	Ccr^*^	0.153	0.057				
	HCRP	0.176	0.028				

Gender and age were forcibly included.

^*^The item by which the biomedical factors' effects on viscera are characterized.

The value of path coefficient was used for standardized estimates.
